# Neuromyelitis optica spectrum disorder patients dyadic coping patterns: a latent profile analysis

**DOI:** 10.3389/fpsyg.2026.1846822

**Published:** 2026-08-14

**Authors:** An-Ning Wang, Zhu-Yun Liu, Hao Liang, Li-Xin Wang, Hao-You Xu, Lin Wei, Xiao-Pei Zhang

**Affiliations:** 1 The Second Clinical College of Guangzhou University of Chinese Medicine, Guangzhou, China; 2 Department of Neurology, The Second Affiliated Hospital of Guangzhou University of Chinese Medicine, Guangdong Provincial Hospital of Chinese Medicine, Guangzhou, China; 3 Department of Nursing, The Second Affiliated Hospital of Guangzhou University of Chinese Medicine, Guangdong Provincial Hospital of Chinese Medicine, Guangzhou, China

**Keywords:** anxiety, associated factors, dyadic coping, healthcare costs, latent profile analysis, neuromyelitis optica spectrum disorders

## Abstract

**Aim:**

This study aims to identify latent subgroups of dyadic coping (DC) patterns among patients with neuromyelitis optica spectrum disorder (NMOSD) and examine factors associated with subgroup classification.

**Design:**

A cross-sectional study.

**Methods:**

The study was conducted from June 2023 to August 2024, enrolling 137 patients with NMOSD. Data were collected via standardized instruments: a demographic questionnaire, the Dyadic Coping Inventory (DCI), the 9-item Patient Health Questionnaire (PHQ-9) for depression screening, and the 7-item Generalized Anxiety Disorder Scale (GAD-7). Latent profile analysis (LPA) was performed using Mplus 8.11 to identify coping subgroups, with distal outcomes (PHQ-9 and GAD-7) examined via the Bolck, Croon, and Hagenaars (BCH) method, followed by multivariate logistic regression to assess subgroup associated factor.

**Results:**

Analysis of the positive and negative coping dimensions revealed three distinct profiles among 137 participants: LP-HN (Low positive-High negative coping, *n* = 22, 16.1%), MP-MHN (Moderate positive-Mild high negative coping, *n* = 46, 33.6%), and BC (Balanced coping, *n* = 69, 50.3%). Significant intergroup differences emerged in premorbid residential status, healthcare payment method, physical activity frequency, the score of PHQ-9 and GAD-7. Multivariate analysis identified healthcare payment method as a significant associated factor. BCH analysis of distal outcomes further demonstrated that the MP-MHN group exhibited the highest levels of anxiety and depression, despite maintaining moderate positive coping.

**Conclusion:**

NMOSD patients demonstrate heterogeneous DC patterns. The healthcare payment method identified as the key associated factor. The MP-MHN subgroup, with the highest psychological burden despite moderate positive coping, warrants prioritized interventions.

## 1 Introduction

Neuromyelitis optica spectrum disorder (NMOSD) is characterized by demyelinating lesions of the central nervous system, with a preference for affecting the optic nerves and the spinal cord, typically resulting in chronic progression (Wingerchuk et al., 2007). The pathogenesis of NMOSD is primarily driven by autoantibodies targeting aquaporin-4 (AQP4), which is a water transport protein highly expressed on the footprints of astrocytes in the central nervous system. Binding of anti-AQP4 immunoglobulin G can trigger complement-dependent cytotoxicity, astrocyte destruction, and subsequent inflammatory demyelination, ultimately leading to neuronal damage (Lennon et al., 2005; Wingerchuk et al., 2015). Global epidemiological studies employing the 2015 International Diagnostic Criteria (IDC) have reported prevalence rates ranging from 0.5 to 10 per 100,000 person-years, with significantly higher rates observed in East Asian populations than in Caucasians and other Asian ethnic groups (Hor et al., 2020). In China, epidemiological surveys estimate an overall NMOSD incidence of 0.278 per 100,000 person-years, with distinct age-related patterns (pediatric: 0.075; adult: 0.347) and notable female predominance (Tian et al., 2020).

Although the pathogenic mechanisms underlying AQP4-seronegative cases are not yet fully elucidated, it has now been established that anti-AQP4 antibodies play a central role in the pathogenesis of most patients (Lennon et al., 2005). In previous studies, the serum positivity rate ranges from 70% to 90% (Hyun et al., 2016; Jarius et al., 2023; Sepúlveda et al., 2018; Villa, 2026). In China, four targeted immunotherapies: eculizumab, inebilizumab, satralizumab, and rituximab, have been officially approved for use in serum-positive NMOSD patients (Kümpfel et al., 2024). However, serum-negative patients or those with unknown antibody status due to limited testing accessibility face uncertainties in diagnosis and treatment, which may become a significant source of severe anxiety. Moreover, all these disease-modifying therapies are extremely expensive. Although national basic medical insurance covers over 95% of the population (Tao et al., 2020), and specific targeted therapies for AQP4-serum-positive NMOSD have been included in the National Reimbursement Drug List since 2023, substantial disparities still exist in reimbursement rates and out-of-pocket costs across regions and individual patients. This economic disparity may exacerbate psychological distress, thereby further aggravating their already existing mental suffering. Clinically, NMOSD is recognized as a relapsing-remitting disorder with substantial disability accumulation (Preziosa et al., 2024), the vast majority of patients exhibit a relapsing course, with each acute episode leading to a stepwise accumulation of neurological deficits. This phenomenon is increasingly recognized as “relapse-associated worsening” (RAW) (Palace et al., 2019). It means that patients experience multidimensional stressors, including disease-related pain, psychological distress (particularly depression and anxiety), and socioeconomic challenges (Beekman et al., 2019; Huang et al., 2020). These compounded burdens substantially impair quality of life and social functioning (Berthele et al., 2023). Moreover, spousal caregivers face comparable psychological burdens while navigating complex treatment decisions and facilitating rehabilitation efforts (Jia et al., 2022).

Dyadic coping (DC) describes the collaborative process through which partners jointly manage stressors, exerting significant influences on mental health, relationship dynamics, and quality of life (Hou et al., 2025). The evidence indicates that effective DC not only enhances marital satisfaction and stability but also promotes psychological well-being and quality of life improvement in couples (Landolt et al., 2023). This construct comprises two dimensions: positive coping (including supportive, delegated, and joint coping) and negative coping (characterized by ambivalent and hostile coping) (Bodenmann, 2005). As a predictive indicator, DC has potential for forecasting mental health trajectories and mitigating emotional disturbances such as depression and anxiety (Johnson et al., 2017). Intervention studies utilizing DC approaches revealed gender-specific patterns, with women exhibiting greater engagement in emotion-focused strategies and stronger associations between positive coping styles and mental health improvements (Forster et al., 2024; Helgeson et al., 2022). The clinical relevance of these findings is accentuated by the female predominance in NMOSD populations. Although the scoring system for DC has a well-defined interpretation range (total scores <111 indicate low level, scores between 111–145 represent moderate level, and scores >145 denote high level), research on DC patterns in NMOSD patients remains severely limited. Moreover, existing assessment methods, which rely excessively on scale scores, may fail to capture complex subtle differences. Latent profile analysis (LPA) provides methodological rigor through data-driven identification of homogeneous subgroups within heterogeneous populations without a prior specified assumption, thereby elucidating latent patterns in complex phenomena. While existing LPA applications in DC research have focused on oncological and reproductive contexts (Tang et al., 2022; Wei et al., 2024; Yan et al., 2024; Zhang et al., 2024), NMOSD populations remain unexplored. This study therefore aimed to investigate heterogeneous DC patterns among NMOSD patients using LPA and identify factors associated with subgroup classification. The aim of this study was to provide a reference for the development of personalized interventions in clinical settings and to increase the quality of care for NMOSD patients. We hypothesized that distinct coping profiles would emerge and that these profiles would be associated with socioeconomic factors and psychological symptoms.

## 2 Materials and methods

### 2.1 Study design and subjects

This single-group cross-sectional study (descriptive design) employed convenience sampling and recruited participants from the neurology outpatient department and inpatient wards of a tertiary hospital in Guangzhou, China, between June 2023 and August 2024. The inclusion criteria included the following: (1) diagnosis of NMOSD per the 2021 Chinese Diagnostic and Therapeutic Guidelines for Demyelinating Optic Neuropathies; (2) age ≥18 years; (3) minimum educational attainment of elementary school; and (4) capacity to provide informed consent with demonstrated cognitive competence for independent questionnaire completion. The exclusion criteria included the following: (1) significant organic pathologies or degenerative joint diseases; (2) pregnancy or lactation; and (3) acute exacerbations of systemic conditions (such as viral encephalitis, systemic lupus erythematosus, rheumatoid arthritis, diabetic ketoacidosis, thyroid storm, acute heart failure, hypertensive crisis, acute leukemia, disseminated intravascular coagulation, acute stroke, or myasthenic crisis).

The sample size was calculated on the basis of the principle of unordered multicategorical multivariate logistic regression, which recommends 10–15 observations per independent variable (Fang, 2012). With 11 independent variables in our study design and an additional 10% allowance for potential questionnaire invalidation, the required sample size ranged from 123 to 184 participants. Based on the sample size requirements for LPA and considerations of model classification accuracy, a minimum sample size of 50 cases per class was necessary to ensure reliable model selection (Nylund et al., 2007). Assuming that the different patterns in this study represent three latent classes, a minimum sample size of 150 cases was required. As this study focused on a rare disease, a total of 137 valid questionnaires were successfully collected and analyzed.

### 2.2 Measures

#### 2.2.1 General information questionnaire

The demographic survey collected sociodemographic characteristics and clinical parameters through two structured modules. The sociodemographic module included sex, age, premorbid occupational status, marital/cohabitation status, premorbid residential status, healthcare payment method, and physical activity frequency. The clinical module evaluated disease duration, relapse frequency, and standardized mental health assessments via the PHQ-9 and GAD-7 scales.

#### 2.2.2 Dyadic coping inventory

The Dyadic Coping Inventory (DCI), originally developed by Bodenmann and Randall, 2012, was cross-culturally adapted to Chinese populations by Xu et al. (2016). This instrument assesses DC strategies categorized into positive (supportive, delegated, and joint coping) and negative (ambivalent and antagonistic coping) dimensions (Johnson et al., 2017). The 37-item scale comprises five domains: stress communication (8 items), supportive coping (10 items), delegated coping (4 items), joint coping (5 items) and negative coping (8 items). Responses were recorded on a 5-point Likert scale (1 = “rarely” to 5 = “very frequently”), with reverse scoring applied to negative coping items. The total scores (range: 35 ∼ 175) were interpreted as follows: < 111 (low coping capacity), 111 ∼ 145 (average range), and > 145 (high coping efficacy), with higher scores indicating stronger positive dyadic functioning. The Cronbach’s α for the scale was 0.774 in this research.

#### 2.2.3 9 -item patient health questionnaire depression module (PHQ-9)

The PHQ-9, a validated depression screening tool developed by Kroenke et al. (2001), evaluates symptom severity through nine items scored on a 4-point scale (0 = “not at all” to 3 = “nearly daily”). The total scores (range: 0 ∼ 27) correspond to the following clinical severity thresholds: 0 ∼ 4 (asymptomatic), 5 ∼ 9 (mild), 10 ∼ 14 (moderate), 15 ∼ 19 (moderately severe), and 20 ∼ 27 (severe). The Cronbach’s α for the scale was 0.892 in this research.

#### 2.2.4 7 -item generalized anxiety disorder scale (GAD-7)

Developed by Spitzer et al. (2006) for anxiety screening, the GAD-7 quantifies symptom severity via seven items on a 4-point response scale (0 = “not at all” to 3 = “nearly daily”). The total scores (range: 0 ∼ 21) are classified as follows: 5 ∼ 9 (mild), 10 ∼ 14 (moderate), and ≥ 15 (severe). Excellent reliability was observed in the current sample (Cronbach’s α = 0.942).

### 2.3 Ethical considerations

This study received ethical approval. All participants provided written informed consent after receiving detailed ethical clearance. The procedures used in this study adhered to the tenets of the Declaration of Helsinki.

### 2.4 Calculation

#### 2.4.1 Data collection

Cross-sectional data were collected during scheduled outpatient revisits or inpatient stays between June 2023 and August 2024 at the Department of Neurology at a tertiary hospital in China. Data collection was conducted following a standardized protocol aligned with preestablished inclusion/exclusion criteria. Participants were systematically recruited from neurology outpatient departments and inpatient units. Trained research staff implemented a three-phase data acquisition process: (1) Researchers provided comprehensive study disclosures, emphasizing voluntary participation and data anonymity; (2) validated questionnaires were administered via a secure digital platform in clinical settings, with real-time completion guidance provided to participants; and (3) incomplete responses were addressed through immediate clarification requests during the completion process. Completed questionnaires were reviewed for missing items, and participants were asked to clarify or complete any omissions on the spot. For participants with visual impairment, research staff provided oral administration of questionnaires or assisted in reading items aloud to ensure accurate completion.

#### 2.4.2 Data analysis

Statistical analyses were performed using Mplus 8.11 and IBM SPSS Statistics 25.0. LPA was conducted in Mplus 8.11 to identify distinct subgroups on the basis of DC scores among NMOSD patients, employing maximum likelihood estimation with robust standard errors (MLR) to address non-normal distributions and full information maximum likelihood (FIML) for missing data handling. Model selection was guided by multiple fit indices: Akaike information criterion (AIC); Bayesian information criterion (BIC); with lower values indicating better fit; entropy, with entropy (range 0–1) closer to 1 indicating clearer classification; and the Lo-Mendell-Rubin (LMR) and Bootstrapped likelihood ratio test (BLRT), where *P* < 0.05 suggested that the K-class model outperformed the (K-1)-class model. Covariates were selected via a two-step process: initially, variables with *P* < 0.05 in univariate analysis were retained, and variables with non-significant univariate results but clear clinical relevance were also included; all candidate variables were then entered into multivariate logistic regression for further screening. Between-group differences in demographic and disease-related characteristics were analyzed in SPSS 25.0 via χ^2^ tests or Fisher’s exact tests, Kruskal–Wallis H tests, and multivariate logistic regression, with statistical significance set at *P* < 0.05. For distal outcomes (PHQ-9 and GAD-7), the Bolck, Croon, and Hagenaars (BCH) three-step procedure was used to compare mean differences across the identified profiles, accounting for classification errors.

## 3 Results

### 3.1 Analysis of general information

We initially enrolled 140 NMOSD patients, 137 of whom completed the questionnaires (retention rate: 97.9%). The cohort comprised 12.41% males (*n* = 17) and 87.59% females (*n* = 120), with a mean age of 42.50 ± 12.39 years. The average DCI score was 111.92 ± 26.11. Detailed demographic and clinical characteristics are summarized in Table 1, and Table 2 shows the five dimensions’ scores of the DCI among different patient subgroups.

**TABLE 1:** The univariate analysis of DC of general information of 137 NMOSD patients.

Variables	Total sample	LP-HN group (*N* = 22)	MP-MHN group (*N* = 46)	BC group (*N* = 69)	χ^2^/H	*P*
Gender					1.599	0.450
Male	17	2 (9.1%)	4 (8.7%)	11 (15.9%)		
Female	120	20 (90.9%)	42 (91.3%)	58 (84.1%)		
Age (years)					0.826	0.662
< 42	74	10 (45.5%)	25 (54.3%)	39 (56.5%)		
> 42	63	12 (54.5%)	21 (45.7%)	30 (43.5%)		
Premorbid occupational status					3.025	0.554
Entered on Physical Labor	48	9 (40.9%)	17 (37.0%)	22 (31.9%)		
Centered on Mental Labor	58	7 (31.8%)	17 (37.0%)	34 (49.3%)		
The else	31	6 (27.3%)	12 (26.1%)	13 (18.8%)		
Marital/cohabitation status					3.943	0.139
With a partner, married	104	14 (63.6%)	33 (71.7%)	57 (82.6%)		
Unmarried, divorced or widowed	33	8 (36.4%)	13 (28.3%)	12 (17.4%)		
Premorbid residential status					13.137	0.003**
Solitary	13	5 (22.7%)	7 (15.2%)	1 (4.5%)		
Living with family	119	16 (72.7%)	37 (80.4%)	66 (95.7%)		
The else	5	1 (4.5%)	2 (4.3%)	2 (2.9%)		
Healthcare payment method					10.375	0.004**
Employee medical insurance	95	16 (72.7%)	32 (69.6%)	47 (68.1%)		
Resident medical insurance	32	4 (18.2%)	7 (15.2%)	21 (30.4%)		
Fully self-financed	10	2 (9.1%)	7 (15.2%)	1 (1.4%)		
Frequency of physical activity					10.421	0.010*
Sometimes	22	2 (9.1%)	13 (28.3%)	7 (10.1%)		
Often	53	8 (36.4%)	12 (26.1%)	33 (47.8%)		
Frequently	62	12 (54.5%)	21 (45.7%)	29 (42.0%)		
Disease duration (years)					5.579	0.233
0∼3	55	8 (36.4%)	23 (50.0%)	24 (34.8%)		
3∼5	26	5 (22.7%)	4 (8.7%)	17 (24.6%)		
≥ 5	56	9 (40.9%)	19 (41.3%)	28 (40.6%)		
Number of relapses (times)					2.958	0.565
0∼3	82	16 (72.7%)	28 (60.9%)	38 (55.1%)		
3∼5	20	2 (9.1%)	8 (17.4%)	10 (14.5%)		
≥ 5	35	4 (18.2%)	10 (21.7%)	21 (30.4%)		
Score of PHQ-9 [Median (Q_1_, Q_3_)]	137	6.5 (3.75, 12.25)	10.5 (6, 14)	8 (3, 12.5)	6.573	0.037*
Score of GAD-7 [Median (Q_1_, Q_3_)]	137	3 (0.75,7)	7.5 (3.75,14)	4 (0.5, 9)	12.649	0.002**

The LP-HN group (Low positive-High negative coping), the MP-MHN group (Moderate positive-Mild high negative coping), the BC group (Balanced coping); DC, dyadic coping; NMOSD, neuromyelitis optica spectrum disorders; P, probability value.

*P < 0.05,

**P < 0.01.

**TABLE 2:** Scores of different subgroups of DC in NMOSD patients.

Dimension	Total (*N* = 137)	LP-HN group (*N* = 22)	MP-MHN group (*N* = 46)	BC group (*N* = 69)
Stress communication	23.32 ± 8.11	9.36 ± 2.15	20.96 ± 4.36	29.33 ± 3.74
Supportive coping	31.0 ± 10.60	12.41 ± 3.55	27.57 ± 4.09	39.39 ± 4.29
Negative coping	30.07 ± 6.06	33.95 ± 4.41	27.89 ± 3.83	30.29 ± 7.04
Delegated coping	12.37 ± 4.01	5.5 ± 1.97	11.80 ± 1.97	14.94 ± 2.18
Joint coping	15.07 ± 5.36	5.82 ± 1.79	13.61 ± 3.09	19.00 ± 2.29
Total score	111.92 ± 26.11	67.05 ± 5.66	101.83 ± 8.80	132.96 ± 11.51

the LP-HN group (Low positive-High negative coping), the MP-MHN group (Moderate positive-Mild high negative coping), the BC group (Balanced coping); DC, dyadic coping; NMOSD, neuromyelitis optica spectrum disorders.

### 3.2 Latent profile classification

A LPA was conducted using the average scores of the DCI dimensions for NMOSD patients as input variables. The analysis considered models ranging from 1 to 5 subgroups, as presented in Table 3.

**TABLE 3:** Model-fitting indices of DC among NMOSD patients.

Category2	K	Log (L)	AIC	BIC	aBIC	P (LMR)	P (BLRT)	Entropy	Class probability (%)				
									1	2	3	4	5
Category1	1	−951.051	1922.103	1951.303	1919.667	–	–	–	100.0				
Category2	2	−753.398	1538.796	1585.515	1534.898	< 0.001	< 0.001	0.961	21.9	78.1			
Category3	3	−656.551	1357.102	1421.342	1351.744	< 0.001	< 0.001	0.945	16.1	33.6	50.3		
Category4	4	−626.665	1309.329	1391.089	1302.509	0.195	< 0.001	0.949	35.7	11.7	13.9	38.7	
Category5	5	−591.206	1250.412	1349.691	1242.130	0.062	< 0.001	0.949	7.3	13.1	31.4	36.5	11.7

AIC, Akaike information criterion; BIC, Bayesian information criterion; aBIC, adjusted Bayesian information criterion; BLRT, bootstrapped likelihood ratio test; LMR, Lomendell-Rubin; DC, dyadic coping; NMOSD, neuromyelitis optica spectrum disorders. Explanation: The smaller the values of the AIC, BIC and aBIC are, the better the model fit, the better it can explain the data and not overfitting. All the models had entropy values exceeding 0.8, reflecting a classification accuracy above 90%. The category 3 model demonstrated lower AIC, BIC and aBIC values, significant LMR and BLRT results (*p* < 0.05), and high entropy. Although categories 4 and 5 showed marginally higher entropy, nonsignificant LMR tests and lower class probabilities compromised model stability and interpretability. On the basis of combined statistical and clinical evaluations, the category 3 model was selected as the best model.

During the model evaluation phase, the values of the fit indices (AIC, BIC, and aBIC) decreased as the number of subgroups increased, indicating that increased model complexity improved model fit. The entropy values also increased with the number of subgroups, and all the models presented entropy values exceeding 0.8, indicating a classification accuracy greater than 90%. When the model included 3 subgroups, the AIC, BIC, and aBIC values were all lower, and both the LMR and BLRT values were significant (*P* < 0.05), whereas the entropy remained relatively high. Although the entropy values for the 4- and 5-subgroup models were slightly higher, the LMR test results were non-significant, and some classes presented low probability proportions, which may compromise model stability and interpretability. After comprehensively evaluating the model fit indices and clinical significance, a 3-subgroup model was selected as the optimal solution.

**FIGURE 1:**
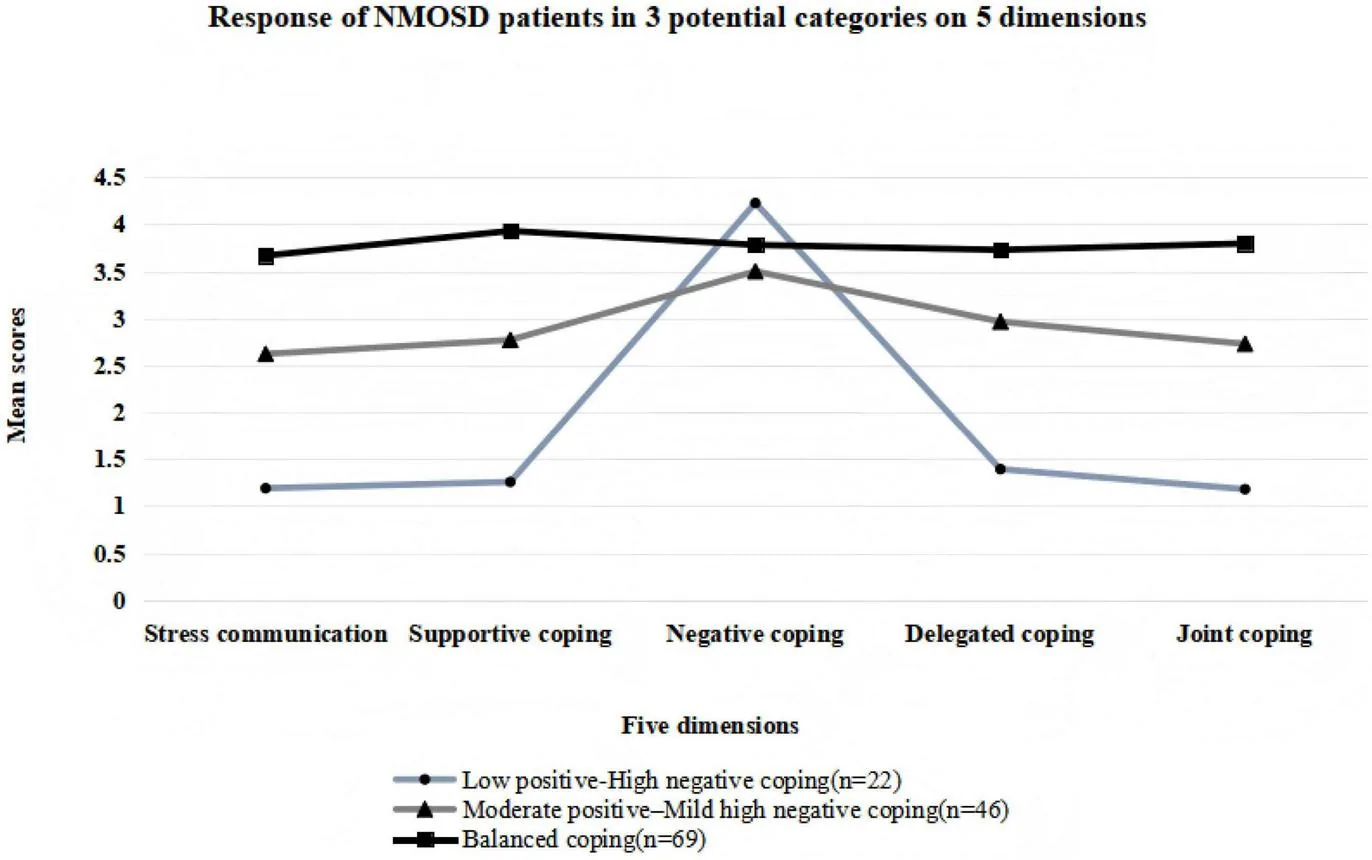
Response of NMOSD patients in 3 potential categories on 5 dimensions.

The characteristics of the subgroups are depicted in Figure 1. Category 1 was characterized by lowest scores on the stress communication, supportive coping, delegated coping, and joint coping dimensions and highest scores on the negative coping dimension. This subgroup was labeled “LP-HN (Low positive-High negative coping) group.” Category 2 was characterized by moderate scores on the stress communication, supportive coping, delegated coping, and joint coping dimensions and mild high scores on the negative coping dimension. This subgroup was labeled “MP-MHN (Moderate positive-Mild high negative coping) group” . Category 3 was characterized by mild high scores on all dimensions, including stress communication, supportive coping, delegated coping, joint coping, and negative coping. This subgroup was labeled “BC (balanced coping) group”.

### 3.3 Sensitivity analysis

Among the three groups identified in this study’s LPA analysis, the LP-HN group and the MP-MHN group exhibited similar trends in positive dimensions (Figure 1). To determine whether these distinctions resulted solely from differences in negative dimension scores, a sensitivity analysis was conducted: another LPA analysis that completely excluded negative dimensions and used only four positive subdimensions (stress communication, supportive coping, delegated coping, and joint coping) as classification criteria. The model selection criteria (listed in Supplementary Table 1) indicate that both Category 2 and Category 3 models are statistically acceptable; however, the entropy value of the Category 2 model is higher than that of the Category 3 model (0.959 vs. 0.945), suggesting that when considering only positive dimensions, the Category 2 structure exhibits clearer classification boundaries.

If Category 1 and Category 2 merely represent a “gradual variation” in negative intensity within the same underlying population, they should be merged into a single category after excluding the negative dimension. However, sensitivity analysis results do not support this hypothesis: Profile 1 from the principal analysis (the LP-HN group, mean across four dimensions ≈1.25) still corresponds to the “low-positive group” in sensitivity analysis (Category 1, mean ≈1.50); whereas Profile 2 from the principal analysis (the MP-MHN group, mean ≈2.77) corresponds to a more positively oriented group with higher mean scores (Category 2, mean ≈3.46). The overall positive score level of this group falls between that of Profile 2 (2.77) and Profile 3 (3.78) in the principal analysis, suggesting that members of the Profile 2 and Profile 3 may exhibit some degree of aggregation in terms of positive dimensions after excluding the negative dimension.

This indicates that although Profile 1 and Profile 2 exhibit similar trends in positive dimensions, they do not merge into a single category when negative dimensions are excluded.

### 3.4 BCH analysis: mental health outcomes

To validate the clinical relevance of the identified coping profiles, we compared the three profiles on depression (PHQ-9) and anxiety (GAD-7) using the BCH three-step procedure. As shown in Table 4, the MP-MHN group exhibited the highest levels of both depression (*M* = 10.096, SE = 0.829) and anxiety (*M* = 8.375, SE = 0.862) among all three profiles. Specifically, the MP-MHN group reported significantly higher anxiety than the LP-HN group (*M* = 4.495, SE = 1.041; χ^2^ = 8.125, *p* = 0.004) and the BC group (*M* = 4.940, SE = 0.561; χ^2^ = 10.663, *p* = 0.001). For depression, the MP-MHN group also scored significantly higher than the BC group (χ^2^ = 6.746, *p* = 0.009), although the difference between the LP-HN group and the MP-MHN group did not reach statistical significance (*p* = 0.211).

**TABLE 4:** Means, standard errors, and pairwise comparisons of distal outcomes across latent profiles.

Variable	Mean ± SE			Pairwise comparisons (χ^2^/P)			Overallχ^2^(P)
	Class 1	Class 2	Class 3	1 vs. 2	1 vs. 3	2 vs. 3	
PHQ-9	8.134 ± 1.316	10.096 ± 0.829	7.269 ± 0.664	1.565 (0.211)	0.344 (0.558)	6.746 (0.009**)	6.768 (0.034*)
GAD-7	4.495 ± 1.041	8.375 ± 0.862	4.940 ± 0.561	8.125 (0.004**)	0.141 (0.707)	10.663 (0.001**)	12.293 (0.002**)

PHQ-9, 9-item Patient Health Questionnaire Depression Module; GAD-7, 7-item Generalized Anxiety Disorder Scale; SE, Standard Errors; Class 1:the LP-HN group (Low positive-High negative coping), Class 2: the MP-MHN group (Moderate positive-Mild high negative coping), Class 3: the BC group (Balanced coping). *P < 0.05, **P < 0.01.

Contrary to the expected outcome, given that the LP-HN group already exhibited highest scores on the negative dimension of the DCI, the study revealed a surprising finding: the MP-MHN group may actually impose the greatest psychological burden (anxiety and depression).

### 3.5 Factors related to different subgroups

As shown in Table 1, significant differences in DC dimension scores were observed across subgroups (all *P*-values < 0.05), indicating heterogeneity in DC patterns among NMOSD patients.

The three latent profiles demonstrated statistically significant differences (all *p* < 0.05) in premorbid residential status, healthcare payment method, physical activity frequency, and scores on the PHQ-9 and GAD-7 scales among patients with NMOSD.

Multivariate logistic regression analyses were employed to evaluate three distinct categories of DC among patients with NMOSD, using the MP-MHN group as the reference. Although disease duration and marital status did not demonstrate statistical significance in the univariate analyses (*p* ≥ 0.05), they were retained in the multivariate model due to their established clinical relevance, ensuring a more comprehensive adjustment for potential confounding factors.

As summarized in Table 5, the final adjusted model identified healthcare payment method as significant associated factor of subgroup membership in DC profiles among NMOSD patients. The payment method for resident medical insurance was significantly associated with the grouping (OR = 19.52, *P* = 0.013), while employee medical insurance also exhibited a marginal association (OR = 8.94, *P* = 0.054).

**TABLE 5:** Results of logistic regressions for the DC subgroups.

Variables		LP-HN group				BC group			
		B	OR	95%CI	*P*	B	OR	95%CI	*P*
Frequency of physical activity	Sometimes	−1.137	0.321	0.059∼1.743	0.188	−0.781	0.458	0.139∼1.509	0.199
	Often	0.245	1.277	0.39∼4.188	0.686	0.695	2.004	0.784∼5.121	0.147
	Frequently (reference)								
Marital/cohabitation status	With a partner, married	−0.493	0.611	0.171∼2.178	0.447	0.177	1.193	0.409∼3.481	0.747
	Unmarried, divorced or widowed (reference)								
Disease duration (years)	0∼3	−0.448	0.639	0.179∼2.276	0.489	−0.167	0.846	0.325∼2.202	0.732
	3∼5	0.953	2.593	0.528∼12.723	0.240	0.905	2.472	0.646∼9.461	0.186
	≥ 5 (reference)								
Premorbid residential status	Solitary	0.537	1.711	0.109∼26.871	0.702	−1.393	0.248	0.012∼4.984	0.363
	Living with family	0.134	1.144	0.089∼14.715	0.918	0.897	2.452	0.29∼20.708	0.410
	The else (reference)								
Healthcare payment method	Employee medical insurance	0.374	1.453	0.227∼9.293	0.693	2.191	8.943	0.965∼82.886	0.054
	Resident medical insurance	0.5	1.648	0.194∼13.966	0.647	2.972	19.523	1.862∼204.684	0.013*
	Fully self-financed (reference)								

OR, odds ratio; CI, confidence interval; P probability value. The reference group is the MP-MHN group (Moderate positive-Mild high negative coping), the LP-HN group (Low positive-High negative coping), the BC group (Balanced coping); DC, dyadic coping .

*P < 0.05.

## 4 Discussion

### 4.1 Heterogeneity exists in the DC of NMOSD patients

LPA identified three clinically relevant categories: the LP-HN (Low positive-High negative coping, *n* = 22, 16.1%) group, the MP-MHN (Moderate positive-Mild high negative coping, *n* = 46, 33.6%) group, and the BC (Balanced coping, *n* = 69, 50.3%) group. These findings are supported by previous DCI-based studies (Jiang et al., 2026; Zhang et al., 2024), which collectively demonstrate that DC is not a unidimensional construct, individuals exhibit heterogeneous patterns across positive and negative coping dimensions rather than a uniform response to disease-related stress, which collectively demonstrate that DC is not a unidimensional construct; rather, individuals exhibit heterogeneous patterns across positive and negative coping dimensions, suggesting that patients facing chronic health stressors do not adopt a uniform coping style. Suggesting that NMOSD patients may develop distinct coping modes influenced by individual psychological resources, family dynamics, and social support. In the LP-HN group, scores of negative dimension were more pronounced, which is consistent with the findings of Meier et al. (2011). This pattern may stem from disease-related pain (Mealy et al., 2020), poor sleep quality (Barzegar et al., 2019), fear of disease uncertainty, inadequate social support, or reduced psychological resilience. These mechanistic insights guide individualized interventions: for the LP-HN group, implementing cognitive restructuring protocols (Bryant et al., 2024; Ciharova et al., 2021) alongside dyadic communication training is recommended; for the MP-MHN group, emotion regulation and distress tolerance training is advised (Norman-Nott et al., 2024); for the BC group, this group exhibits high scores on both the positive and negative dimensions simultaneously. This “dual-high” pattern conceptually parallels the coexistence pattern described in Oh and Tong’s Mixed Emotion Specificity Framework (Oh and Tong, 2022): when individuals hold conflicting evaluations of similar intensity, it generates a dual effect—inducing internal conflict (such as relationship tension or behavioral counterbalancing) while also stimulating integrative thinking and enhancing cognitive flexibility. It is important to note that this framework primarily focuses on cognitive evaluation, whereas the BC’s “dual-high” manifestation reflects coexisting behavioral strategies, suggesting the group may operate in a state of “high engagement and high consumption” —actively mobilizing support resources yet struggling to suppress negative reactions. Therefore, it is recommended to implement a comprehensive intervention program for this group that integrates two complementary components. First, conduct structured joint problem-solving training to enhance partners’ ability to communicate stress effectively and collaborate on resolving issues, transforming generalized support-seeking behaviors into concrete, actionable collaborative actions, thereby mitigating the impact of stress on the relationship. Evidence from couples with high functioning but experiencing stress indicates that such interventions significantly improve relationship satisfaction and dual coping skills (Zemp et al., 2017), which aligns well with the baseline characteristics of the BC group, highly positive coping patterns alongside high negative coping tendencies but without clinical depression, suggesting that behavioral skill reinforcement is more appropriate than symptom relief alone. Second, incorporate coping flexibility training to help individuals identify contexts triggering negative coping strategies and apply cognitive restructuring instead of habitual blame or avoidance; meta-analysis evidence demonstrates a positive correlation between coping flexibility and improvements in both positive and negative adaptation (Chen et al., 2025). This dual-track strategy transforms the characteristic of “coexistence of contradictions” in the BC group into a more adaptive stress-buffering mechanism. This approach underscores the necessity of acknowledging patient heterogeneity in clinical practice and tailoring interventions to specific patient subgroups.

### 4.2 Sensitivity analysis

In sensitivity analysis focusing solely on positive dimensions, we found that although Profile 1 and Profile 2 exhibit similar trends in positive dimensions, they do not merge when negative dimensions are excluded, with the high-positive group mean (3.46) falling between Profile 2 (2.77) and Profile 3 (3.78). This pattern indicates two clinically relevant insights.

First, the non-merger of Profile 1 and Profile 2 confirms that their distinction is not artificially driven by negative coping, but reflects inherent differences in positive dimensions themselves. Second, the intermediate position of the sensitivity-derived high-positive group (3.46) reveals that excluding negative dimensions causes moderate-to-high positive individuals to aggregate into a broad cluster; reintroducing negative dimensions splits this cluster into Profile 2 and Profile 3. Crucially, despite similar positive levels, their negative score difference (≈3.5 vs. ≈3.7) suffices to mark a risk-prone coping pattern, demonstrating that the negative dimension refines risk stratification among “non-low positivity” individuals. We argue this constitutes the statistical and clinical rationale for the three-model over the two-model approach.

In conclusion, sensitivity analysis not only did not undermine the three-model structure but also confirmed that positive dimensions have basic discriminatory power, while negative dimensions enable finer clinical subgrouping, together providing a more detailed and clinically meaningful perspective.

### 4.3 Distal outcomes of coping profiles: differences in mental health outcomes

The BCH analysis further validates the clinical distinctiveness of the three coping profiles. the MP-MHN group exhibited the highest psychological burden (anxiety and depression) despite maintaining moderate levels of positive coping; this pattern was surprising. Unlike the LP-HN group, which showed low engagement across positive coping dimensions (suggesting possible emotional disengagement from relationship efforts), the MP-MHN group maintained moderate positive engagement while simultaneously exhibiting elevated negative coping. This combination may create a cycle of frustrated efforts and relational strain, paradoxically generating the greatest psychological distress. This finding aligns with the stress spillover effect (Miyata et al., 2022), where interpersonal stress transmission between partners undermines DC capacity and emotional regulation. Notably, the MP-MHN group’s moderate coping style, neither highly adaptive nor severely dysfunctional, may render these individuals particularly vulnerable to contextual stressors and relational strain, as they lack the protective factors of balanced coping but remain sufficiently engaged to experience the emotional costs of negative interactions.

Given that the MP-MHN group exhibited the highest psychological burden despite moderate positive engagement, interventions for this subgroup should prioritize reducing the incongruence between their continued relationship efforts and negative interaction patterns. Emotionally Focused Couple Therapy may help break negative interaction cycles while strengthening secure attachment (Johnson and Greenman, 2006). Additionally, Acceptance and Commitment Therapy (ACT) tailored for NMOSD has demonstrated significant improvements in depression and anxiety in this population (Esiason et al., 2025). For this group’s heightened emotional distress, online Dialectical Behavioral Therapy (DBT) skills training targeting emotion dysregulation may also be beneficial (Norman-Nott et al., 2025). Collectively, these approaches suggest that the MP-MHN group patients may benefit most from interventions that simultaneously address emotion regulation at the individual level and communication patterns at the dyadic level.

### 4.4 Factors affecting DC in NMOSD patients

The finding that medical insurance was the only significant predictor of class membership may be interpreted in light of socioeconomic factors that are related to coping resources. This significant association may be explained by better access to healthcare services and a reduction in financial strain, which are factors that have been linked to greater resource availability for effective DC by ensuring greater resource availability (Angelelli et al., 2022; Powell-Wiley et al., 2022). This mechanism is further substantiated by Liao’s health economics model (Liao et al., 2023), which confirms that the financial protection afforded by insurance increases routine healthcare utilization while shielding households from catastrophic expenditures, pathways that may contribute to more adaptive coping through key socioeconomic pathways. As noted in the Introduction, although China has achieved near-universal health insurance coverage and has recently included several targeted NMOSD therapies in the national drug list, the actual financial burden experienced by patients remains substantial. In China, basic medical insurance covers over 95% of the population. Specifically, a recent cross-sectional survey of 375 Chinese NMOSD patients conducted between November 2022 and January 2023 found that 96.80% of them had some type of National Basic Medical Insurance (NBMI) (Yu et al., 2024). Moreover, NMOSD was included in the first batch of China’s Rare Disease List in 2018 (National Health Commission of the People’s Republic of China, 2018). In recent years, targeted therapies such as inebilizumab and satrelizumab have also been successively added to the national medical insurance catalog. However, a significant gap remains between nominal medical insurance coverage and actual financial burdens. Previous survey data on Chinese NMOSD patients indicate that 88.1% of respondents still reported insufficient medical insurance coverage to cover all disease-related expenses (Huang et al., 2020). Another study based on nationwide medical insurance claims data found that even after reimbursement, patients still face out-of-pocket expenses averaging 2,787 yuan annually (Wu et al., 2022). These figures demonstrate that although universal coverage provides a safety net, the out-of-pocket burden, particularly for expensive targeted immunotherapies, remains substantial for many families. In contrast, particular attention must be directed toward patients facing substantial out-of-pocket payments, as their elevated financial stress is consistently correlated with poorer coping profiles (Bannon et al., 2021; Knapp et al., 2022). This association often coexists with increased family conflict, emotional exhaustion, and a noticeable erosion of social support networks.

Therefore, health insurance transcends its primary role as a payment mechanism and emerges as a critical social factor of health, profoundly influencing psychosocial adaptation and family functioning. To systematically address patient resilience and quality of life, future chronic disease management strategies may benefit integrating financial risk protection into holistic care frameworks. This is particularly vital for designing targeted support policies for vulnerable populations burdened by high out-of-pocket costs, with the goal of addressing socioeconomic disparities that undermine coping capacity. However, the wide confidence intervals observed for these estimates warrant cautious interpretation, and this finding should be considered preliminary pending replication in larger samples. Future research with more balanced samples is needed to confirm these associations and further explore the pathways through which socioeconomic factors may related to DC in NMOSD patients.

## 5 Enlightenment for clinical practice

In clinical practice, this study supports the implementation of tailored interventions for NMOSD patients on the basis of their identified coping styles. A tiered intervention framework should be adopted, with early implementation of structured strategies being critical for optimal outcomes. Given that BCH analysis has confirmed significant differences in psychological burden among the three groups, early implementation of structured interventions is crucial for optimizing prognosis. For example, integrating the GAD-7 and PHQ-9 scale into outpatient follow-up protocols can systematically screen for psychological burden, especially for the MP-MHN group, while incorporating the biopsychosocial-spiritual model into a comprehensive assessment framework enhances multidimensional evaluation (Chan et al., 2025; Shi et al., 2023).

Clinical nursing practice should incorporate systematic assessment of patients’ financial situations and health insurance coverage, with particular attention to those facing high out-of-pocket medical expenditures. Proactive communication regarding insurance policies, planned treatment pathways, and available community resources (such as chronic disease management programs) can help stabilize psychological well-being indirectly, reduce treatment interruptions linked to cost-related concerns, and foster the use of adaptive coping strategies. Furthermore, emphasizing evidence-based benefits of treatment, including reduced recurrence risks, may enhance patient adherence and support the shift toward more proactive coping profiles.

## 6 Limitations

First, this study exclusively enrolled patients with neuromyelitis optica spectrum disorders (NMOSD) recruited from the neurology outpatient department and inpatient wards of a tertiary hospital in Guangzhou, employing convenience sampling methods, which may introduce selection bias, and it remains unclear whether the conclusions can be generalized to other healthcare settings, geographic regions, or populations with different demographic and insurance characteristics. For instance, the observed associations may vary in primary care facilities, centers with different referral systems, or countries implementing universal health coverage or having distinct insurance frameworks. Second, the sample size was relatively small (*N* = 137), particularly for LPA, where a minimum of 50 cases per subgroup are typically recommended to ensure robust parameter estimation; given that NMOSD is a rare disease, the present sample failed to meet this criterion. This directly resulted in overly wide confidence intervals for key regression estimates (such as health insurance variables), unstable point estimates, and reduced reliability. Therefore, we advise readers to interpret the effect sizes cautiously and consider the results as exploratory rather than definitive conclusions. Although the LPA model demonstrated good fit indices and passed robustness tests, its findings require validation in larger cohorts. Third, this study collected data only from patients themselves, without obtaining corresponding reports from their spouses or other family caregivers. Consequently, we were unable to examine interactive effects, and our findings reflect only the patient’s subjective perspective. The study design did not account for potential confounding variables (such as patient-caregiver interactions). Fourth, While the analysis revealed static associations between associated factors and coping patterns, causal relationships were not established, nor was dynamic evolution over time tracked. Future studies should adopt longitudinal designs with regular assessments. Fifth, this study did not incorporate key covariates such as spouse mental health and social support networks, nor did it conduct a systematic evaluation of visual impairment, a condition frequently observed in NMOSD patients, since visual status may significantly influence medication adherence, healthcare-seeking behavior, and self-reported outcomes. The absence of these variables may introduce unmeasured confounding or effect modification, limiting a comprehensive understanding of the role of family systems and compromising the robustness assessment of core associations. Sixth, this study did not perform serological testing for AQP4 antibodies, making it impossible to conduct subgroup analysis based on serological status. Since AQP4-IgG seropositivity may influence disease severity, recurrence frequency, and treatment response, the absence of this stratification limited our ability to examine differences in coping patterns among distinct immune subtypes. Additionally, the study did not include healthy individuals or patients with other neurological or chronic diseases as controls, thus preventing direct comparison of anxiety levels and coping strategies between NMOSD patients and the general population or other disease groups. Subsequent studies should expand sample sizes to confirm the stability and generalizability of these categories, consider methodological approaches like multicenter designs and actor-partner interdependence models, incorporate serological stratification (such as AQP4-IgG status) to enable subgroup analyses, and include control groups of healthy individuals or patients with other chronic conditions to clarify whether the observed coping patterns and anxiety levels are disease-specific or shared across disorders, and incorporate policy-relevant factors (such as regional disparities in health insurance coverage). The use of mixed-method strategies (combining quantitative longitudinal tracking and qualitative interviews) can provide deeper insights into context-specific coping strategies across socioeconomic backgrounds, thereby informing targeted intervention development.

## 7 Conclusion

LPA identified three distinct DC patterns among patients with NMOSD: the LP-HN group (Low positive-High negative coping, *n* = 22, 16.1%), the MP-MHN group (Moderate positive-Mild high negative coping, *n* = 46, 33.6%) and the BC group (Balanced coping, *n* = 69, 50.3%). The analysis confirmed significant heterogeneity between these subgroups, with the health medical insurance identified as key associated factors. Notably, distal outcomes conducted through BCH analysis further revealed that the MP-MHN group exhibited the highest levels of psychological burden (anxiety and depression) despite maintaining a moderate level of positive coping strategies, underscoring the clinical urgency of implementing interventions for this subgroup. These findings offer a structured framework for developing personalized nursing interventions aimed at enhancing DC. Implementing these interventions through stratified, profile-specific strategies is recommended for optimal effectiveness.

## Data Availability

The raw data supporting the conclusions of this article will be made available by the authors, without undue reservation.
