# Supplementary material for: Neuromyelitis optica spectrum disorder patients dyadic coping patterns: a latent profile analysis
Source: Front Psychol. 2026 Aug 14;17:1846822. doi: 10.3389/fpsyg.2026.1846822 (PMC13521793; doi:10.3389/fpsyg.2026.1846822)
Supplement: Supplementary file 1 [file Supplementary_file_1.docx]

| Categories | K | Log（L） | AIC | BIC | aBIC | P(LMR) | P(BLRT) | Entropy | Size of smallest class(%) |
| --- | --- | --- | --- | --- | --- | --- | --- | --- | --- |
| Category1 | 1 | -795.313 | 1606.627 | 1629.986 | 1604.678 | — | — | — | 100.0 |
| Category2 | 2 | -600.927 | 1227.855 | 1265.815 | 1224.688 | <0.001 | <0.001 | 0.959 | 21.9 |
| Category3 | 3 | -507.595 | 1051.190 | 1103.750 | 1046.806 | <0.001 | <0.001 | 0.945 | 16.8 |
| Category4 | 4 | -482.616 | 1011.232 | 1078.392 | 1005.630 | 0.065 | <0.001 | 0.956 | 7.3 |
| Category5 | 5 | -456.191 | 968.383 | 1050.142 | 961.563 | 0.146 | <0.001 | 0.952 | 6.6 |

**Supplementary Table 1 LPA fit indices based on positive coping subdimensions only**

Note:  **LPA,** latent profile analysis **; AIC**, Akaike information criterion; **BIC**, Bayesian information criterion; **aBIC**, adjusted Bayesian information criterion; **BLRT**, bootstrapped likelihood ratio test; **LMR**, Lomendell-Rubin.
